# Maternal serum alpha-1 antitrypsin levels in spontaneous preterm and term pregnancies

**DOI:** 10.1038/s41598-024-61206-z

**Published:** 2024-05-11

**Authors:** Pinja Tissarinen, Heli Tiensuu, Antti M. Haapalainen, Eveliina Ronkainen, Liisa Laatio, Marja Vääräsmäki, Hanna Öhman, Mikko Hallman, Mika Rämet

**Affiliations:** 1 Research Unit of Clinical Medicine and Medical Research Center, Department of Pediatrics and Adolescent Medicine, Oulu University Hospital, University of Oulu, PO Box 5000, 90014 Oulu, Finland; 2 Research Unit of Clinical Medicine, Department of Obstetrics and Gynecology, Oulu University Hospital, University of Oulu, Oulu, Finland; 3 Research Unit of Clinical Medicine and Medical Research Center, Department of Obstetrics and Gynecology, Oulu University Hospital, University of Oulu, Oulu, Finland; 4 Faculty of Medicine, Biobank Borealis of Northern Finland, Oulu University Hospital, University of Oulu, Oulu, Finland; 5 Faculty of Medicine and Health Technology, Tampere University, Tampere, Finland

**Keywords:** Paediatric research, Preterm birth

## Abstract

Currently, there are no accurate means to predict spontaneous preterm birth (SPTB). Recently, we observed low expression of alpha-1 antitrypsin (AAT) in SPTB placentas. Present aim was to compare the concentrations of maternal serum AAT in pregnancies with preterm and term deliveries. Serum C-reactive protein (CRP) was used as a reference inflammatory marker. Two populations were studied. The first population comprised women who eventually gave birth spontaneously preterm (SPTB group) or term (control group). The second population included pregnant women shortly before delivery and nonpregnant women. We observed that serum AAT levels were higher in the SPTB group than in the controls, and a similar difference was observed when serum CRP was considered in multivariable analysis. However, the overlap in the AAT concentrations was considerable. No statistical significance was observed in serum AAT levels between preterm and term pregnancies at delivery. However, AAT levels were higher at delivery compared to nonpregnant controls. We did not observe a strong correlation between serum AAT and CRP in early pregnancy samples and at labor. We propose that during early pregnancy, complicated by subsequent SPTB, modest elevation of serum AAT associates with SPTB.

## Introduction

Preterm birth, defined as a live birth before 37 completed weeks of pregnancy, is the leading cause of neonatal mortality and morbidity^1,2^. Spontaneous preterm birth (SPTB) is the spontaneous initiation of labor with or without intact fetal membranes^2^. SPTB accounts for nearly 70% of preterm births, and the remaining proportion comprises preterm births without the spontaneous onset of labor (e.g., due to preeclampsia)^3^. Prediction and prevention of SPTB is a challenge in antenatal care^4^. Despite the known risk factors for preterm birth, approximately 40–50% of all SPTBs occur in low-risk pregnancies and have no known etiology^3^. A previous history of preterm birth is the most important pre-pregnancy risk factor^4^. A few tests, including fetal fibronectin measured from the cervicovaginal fluid, are in clinical use as predictive biochemical tests for imminent SPTB^5^. Presumably, mechanical disruption of the placental structures may cause secretion of fetal fibronectin to the cervicovaginal fluid^5^. However, there are no accurate tests available for prediction of SPTB in early pregnancy.

Recently, we observed that low expression of alpha-1 antitrypsin (AAT) on the maternal side of the placenta was associated with SPTB^6^. We proposed that placental AAT participates in maintaining pregnancy, and dysfunctional proteins or low concentrations of placental AAT could predispose mothers to SPTB. Furthermore, we speculated that the low concentration of or dysfunctional AAT could lead to increased degradation of placental fibrinoid components, such as fetal fibronectin, potentially leading to SPTB^6^. AAT is an acute phase protein and a protease inhibitor. It is produced mainly by hepatocytes^7^, but synthesis of AAT has been detected in other cells, such as monocytes and trophoblasts^8^. Serum AAT level increases four to six folds during pregnancy, thus insufficient AAT increase could predispose to adverse pregnancy outcomes^9,10^. AAT deficiency has been suggested to contribute to some extent in a few pregnancy complications, such as preterm birth, spontaneous abortions^9^, and preeclampsia^10^.

Besides AAT, serum C-reactive protein (CRP) is an acute phase protein produced by hepatocytes. CRP is a widely used biomarker of inflammation^11^. CRP levels increase during pregnancy^12,13^. Several studies have proposed CRP as a biomarker for SPTB and other pregnancy complications^14^, but the results have been inconsistent^15–17^. Thus, it is feasible that CRP is not specific enough by itself to use as a predictive test for SPTB. However, better understanding of these inflammatory markers, such as AAT and CRP, at early pregnancy could provide more knowledge about the complex mechanisms leading to SPTB^13^.

The present objective was to establish AAT levels in maternal serum at early pregnancy in relation to pregnancy outcome, spontaneous preterm delivery. Additionally, maternal serum AAT levels were examined at delivery in pregnancies with SPTB, elective preterm birth or term birth, and in serum from nonpregnant participants. Relationship between these acute-phase serum proteins, AAT, and CRP, was also investigated.

## Results

### Maternal serum AAT levels in the first and early second trimesters of the pregnancy

The clinical characteristics of study population 1 are presented in Table 1. A statistically significant difference was observed in some baseline characteristics: parity, a history of preterm births, interpregnancy interval less than 2 years, in vitro fertilization, smoking during pregnancy, and maternal age at sampling (Table 1). Gestational age (GA) at sampling was statistically different between the groups in samples from the first trimester (Table 1). Otherwise, GA at sample collection did not show statistically significant difference between the groups.

**Table 1:** Clinical characteristics of study population 1 (*n* = 529).

Variable	Spontaneous preterm birth	Term birth	*P* value^a^
*n*	131 (24.8)	398 (75.2)	
GA at sampling < 13 + 0	101 (23.1)	337 (76.9)	
GA at sampling 13 + 0–26 + 6	30 (33.0)	61 (67.0)	
GA at sampling (all samples), weeks	10.6 (9.43–12.6)	10.9 (10.1–11.9)	0.126
GA < 13 + 0, weeks	10.1 (9.2–10.9)	10.7 (10.0–11.3)	< 0.001
GA 13 + 0–26 + 6, weeks	15.0 (13.5–16.9)	14.4 (13.6–15.9)	0.380
Parity			< 0.001
Nullipara and primipara	89 (70.1)	142 (40.1)	
Multipara	38 (29.9)	212 (59.9)	
History of preterm births, yes	16 (13.6)	2 (0.6)	< 0.001
History of miscarriage, yes	28 (23.3)	95 (28.9)	0.282
Interpregnancy interval < 2 years, yes	25 (22.5)	152 (45.9)	< 0.001
Primary diseases^b^, yes	17 (13.0)	42 (10.6)	0.522
Pregnancy			
In vitro fertilization or treated with hormones, yes	9 (7.3)	6 (1.7)	0.005
Smoking during pregnancy, yes	14 (17.7)	21 (6.7)	0.004
BMI before pregnancy, kg/m^2^	23.3 (20.4–27.0)	22.8 (20.8–25.8)	0.714
Age of the mother at collection, years	28.9 (26.5–32.9)	30.4 (26.7–34.8)	0.005
Delivery			
GA at birth, weeks	30.6 (28.6–33.6)	40.1 (39.3–40.9)	< 0.001
Onset of delivery			< 0.001
Spontaneous	131 (100.0)	322 (81.3)	
Medically indicated^c^	0 (0.0)	74 (18.7)	
Mode of delivery			< 0.001
Vaginal delivery	92 (74.8)	314 (94.6)	
Cesarean section^d^	31 (25.2)	18 (5.4)	
Preeclampsia, yes	0 (0.0)	3 (0.9)	0.566
Birth weight, g	1505 (1125–2000)	3630 (3350–3940)	< 0.001

*GA* gestational age, *BMI* body mass index.

Continuous variables are displayed as medians (interquartile range [IQR]). Categorical variables are displayed as numbers (%).

^a^*P* value calculated with Mann–Whitney* U* test for continuous variables and with chi-square test for categorical variables.

^b^Primary diseases include cardiovascular diseases, endocrine diseases, pulmonary diseases, gastrointestinal diseases, mental disorders, and rheumatic diseases.

^c^Induction of labor medically: oxytocin, Foley balloon, amniotomy and/or a cesarean section.

^d^Indications for cesarean section were breech position, fetal macrosomia, fear of childbirth, and other fetal or maternal conditions.

**Figure 1:**
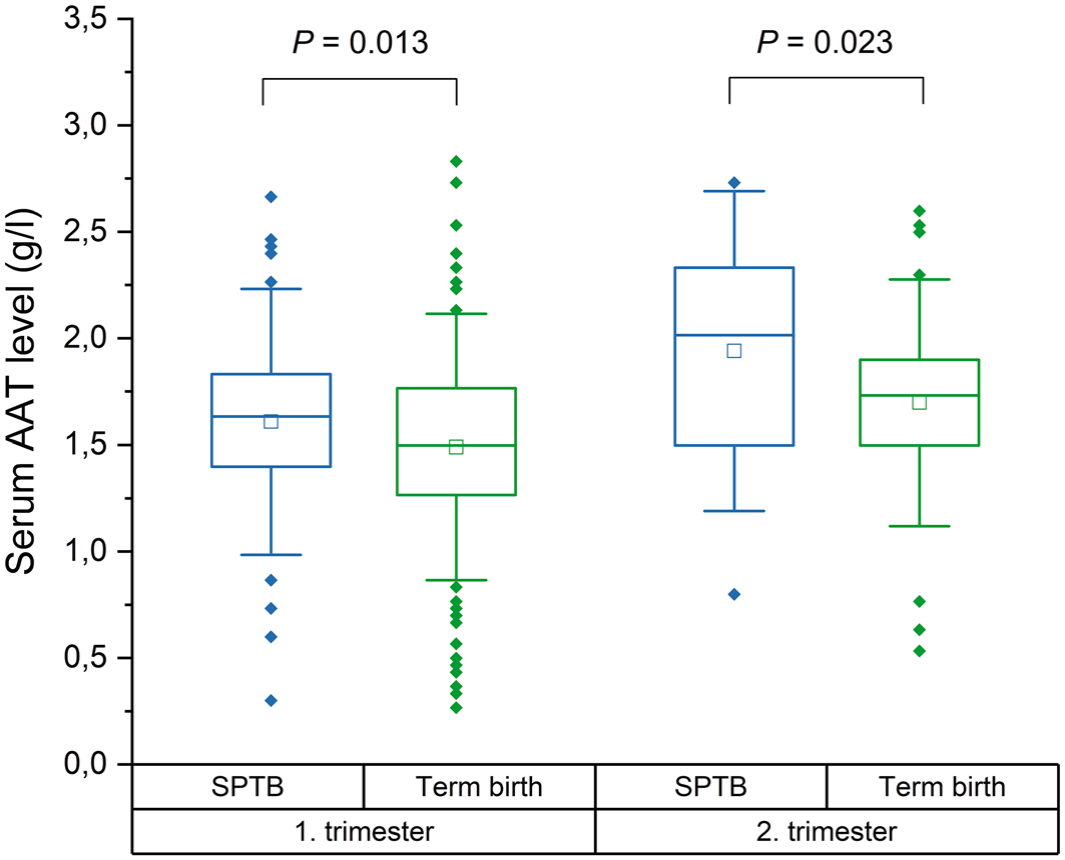
Maternal serum AAT levels in the first and second trimesters of pregnancies resulting in spontaneous preterm birth (SPTB) or term birth (study population 1). Quartiles (25th and 75th) are represented by box and 1.5*SD by whiskers. Inside the box, the band indicates the median, and the mean is indicated with a square. Statistical analysis was performed with Student’s *t*-test.

Generally, AAT levels were higher in the SPTB group than in the control group (both trimesters: the SPTB group mean 1.68 g/l [SD 0.46 g/l] vs. the control group mean 1.52 g/l [SD 0.42 g/l], *P* < 0.001). In the first trimester, the mean AAT level was 1.61 g/l (SD 0.42 g/l) in the SPTB group and 1.49 g/l (SD 0.42 g/l) in the control group (*P* = 0.013, Fig. 1). In the second trimester, the mean AAT concentration was 1.94 g/l (SD 0.50 g/l) in the SPTB group and 1.70 g/l (SD 0.39 g/l) in the control group (*P* = 0.023, Fig. 1).

Effect of in vitro fertilization, smoking during pregnancy, and age of the mother at sampling on serum AAT levels were studied as those were significantly different between the SPTB and control groups (Table 1) and may have an effect on the serum concentration of AAT. Participants in the SPTB group were slightly younger than the controls (Table 1). Very few of the participants were less than 20 years old (*n* < 5), thus effect of young maternal age on serum AAT levels could not be assessed. In vitro fertilization/hormonal treatments, smoking during pregnancy, or advanced maternal age (≥ 40 years, *n* = 30) did not affect serum AAT levels (Supplementary Table S1).

As shown in Fig. 1, some of the participants had low serum AAT levels. A total of 39 participants (the SPTB group *n* = 6, the control group *n* = 32) had AAT level less than 0.96 g/l (the lower limit of normal range of serum AAT according to the HUSLAB Laboratory). After exclusion of those with low AAT concentration, AAT levels were as follows (both trimesters): the SPTB group mean 1.73 g/l (SD 0.40 g/l) vs. the control group mean 1.60 g/l (SD 0.33 g/l), *P* < 0.001. In the first trimester, the mean AAT level was 1.66 g/l (SD 0.35 g/l) and 1.57 g/l (SD 0.32 g/l) in the SPTB and control groups, respectively (*P* = 0.027, Supplementary Fig. S1). Likewise, in the second trimester, the AAT level was higher in the SPTB group compared to the controls (the SPTB group mean 1.98 g/l [SD 0.46 g/l] vs. the control group mean 1.75 g/l [SD 0.31 g/l], *P* = 0.007, Supplementary Fig. S1).

### Maternal serum AAT levels during labor and in pregnant versus nonpregnant participants

**Figure 2:**
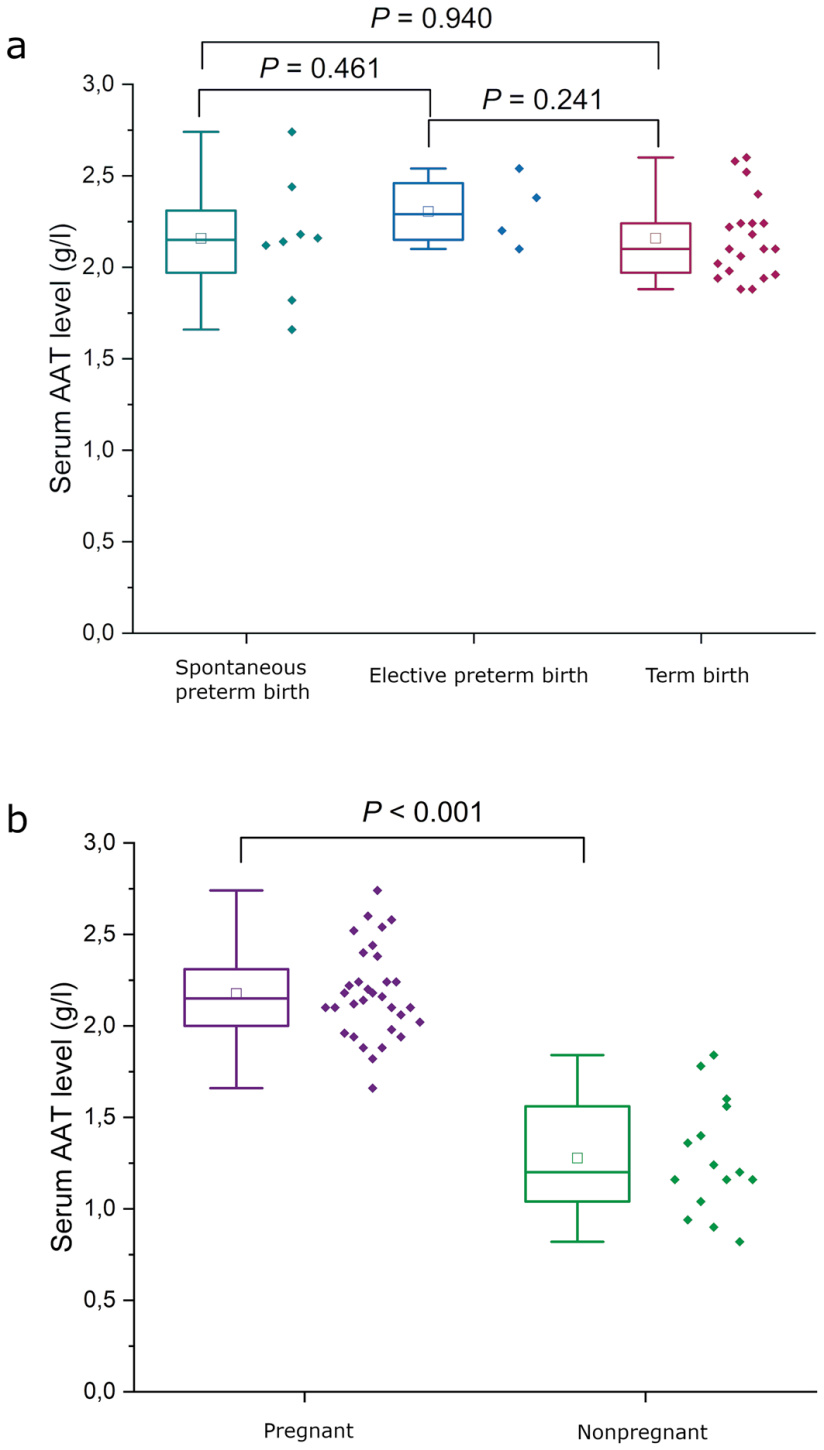
Maternal serum alpha-1 antitrypsin (AAT) levels at labor (study population 2). (**a**) Pregnancy groups (spontaneous preterm birth, elective preterm birth, and term birth) comparable to each other (nominal association). (**b**) Pregnant women (SPTBs, elective preterm births, and term births) compared to nonpregnant participants. Quartiles (25th and 75th) are represented by box and minimum and maximum values by whiskers. Inside the box, the band indicates the median, and the mean is indicated with a square. Statistical analysis was performed with the Mann–Whitney *U* test.

The clinical characteristics of study population 2 are presented in Table 2. Median GAs at sampling were 34.6 weeks in SPTBs, 34.0 weeks in elective preterm births, and 39.7 weeks in term births (Table 2). We did not observe a difference in AAT levels prior delivery between preterm and term deliveries (Fig. 2a). More specifically, medians of maternal serum AAT levels were as follows: SPTBs 2.15 g/l (IQR 1.90–2.38 g/l), elective preterm births 2.29 g/l (IQR 2.13–2.50 g/l), and term births 2.10 g/l (IQR 1.97–2.24 g/l). When we pooled the pregnancy groups together and compared serum AAT levels to nonpregnant participants, the median AAT level was 2.15 g/l (IQR 1.99–2.35 g/l) among the pregnant participants and 1.20 g/l (IQR 1.04–1.56 g/l) in the nonpregnant controls. A statistically significant difference was observed between pregnant and nonpregnant participants (*P* < 0.001, Fig. 2b).

**Table 2:** Clinical characteristics of study population 2 (*n* = 47).

Variable	Spontaneous preterm birth	Elective preterm birth	Term birth	*P* value^a^	Nonpregnant
*n*	8	4	20		15
Parity				0.731	
Nullipara and primipara	6 (75.0)	2 (50.0)	14 (70.0)		N/A
Multipara	2 (25.0)	2 (50.0)	6 (30.0)		N/A
History of preterm births, yes	1 (12.5)	1 (25.0)	1 (5.0)	0.310	N/A
Interpregnancy interval < 2 years, yes	1 (12.5)	0 (0.0)	0 (0.0)	0.375	N/A
Primary diseases^b^, yes	5 (62.5)	3 (75.0)	6 (30.0)	0.147	N/A
Pregnancy					
In vitro fertilization or treated with hormones, yes	2 (25.0)	0 (0.0)	0 (0.0)	0.073	N/A
Smoking during pregnancy, yes	1 (12.5)	1 (33.3)	1 (6.3)	0.327	N/A
BMI before pregnancy, kg/m^2^	24.1 (21.6–26.2)	22.3 (21.0–28.7)	22.5 (20.0–26.8)	0.776	N/A
Delivery					
Age of the mother, years	31.0 (30.0–33.5)	26.5 (23.5–38.5)	31.0 (28.0–33.0)	0.602	27.0 (24.0–33.0)
GA at sampling/birth, weeks	34.6 (31.3–35.4)	34.0 (28.0–35.1)	39.7 (38.9–40.4)	< 0.001	N/A
Onset of delivery				< 0.001	
Spontaneous	8 (100.0)	0 (0.0)	17 (85.0)		N/A
Medically indicated^c^	0 (0.0)	4 (100.0)	3 (15.0)		N/A
Mode of delivery				< 0.001	
Vaginal delivery	8 (100.0)	0 (0.0)	20 (100.0)		N/A
Cesarean section^d^	0 (0.0)	4 (100.0)	0 (0.0)		N/A
Preeclampsia, yes	0 (0.0)	1 (25.0)	0 (0.0)	0.125	N/A
Infection at labor^e^, yes	1 (12.5)	1 (25.0)	0 (0.0)	0.133	N/A
Birth weight, g	2383 (1805–2781)	2410 (1030–3790)	3445 (3147–3833)	0.002	N/A

*N/A* not applicable, *GA* gestational age, *BMI* body mass index.

Continuous variables are displayed as medians (interquartile range [IQR]). Categorical variables are displayed as numbers (%).

^a^*P* value calculated with Kruskal–Wallis *H* test for continuous variables and with with chi-square test for categorical variables.

^b^Primary diseases include cardiovascular diseases, pulmonary diseases, gastrointestinal diseases, mental disorders, and endocrine disorders.

^c^Induction of labor medically: oxytocin, Foley balloon, amniotomy and/or cesarean section.

^d^Indications for cesarean section were breech position, chorioamnionitis, fear of childbirth, and other fetal or maternal conditions.

^e^Infection at labor was determined as clinical signs and symptoms of infection: fever and a significant increase in infection parameters.

Lastly, we illustrated serum AAT levels between the groups (nonpregnant, the first trimester, the second trimester, and women at labor) from both study populations. As seen in Supplementary Fig. S2, the median of serum AAT level seems to have an increasing trend toward the end of pregnancy.

### Correlation between serum AAT and CRP and their effect on SPTB

When including both the first and the second trimesters, the serum AAT level had a positive correlation with serum CRP in both SPTB (*r*_*s*_ = 0.42, *P* < 0.001, Supplementary Fig. S3a) and control (*r*_*s*_ = 0.36, *P* < 0.001, Supplementary Fig. S3b) groups. In the first trimester, the correlations were as follows: the SPTB group *r*_*s*_ = 0.31 (*P* = 0.002, Supplementary Fig. S3c) and the control group *r*_*s*_ = 0.36 (*P* < 0.001, Supplementary Fig. S3d). In the second trimester, a positive correlation was observed in the SPTB group (*r*_*s*_ = 0.79, *P* < 0.001, Supplementary Fig. S3e), whereas the correlation was weaker in the control group (*r*_*s*_ = 0.36, *P* = 0.004, Supplementary Fig. S3f). We observed no correlation between maternal serum AAT and CRP during labor (*r*_*s*_ = − 0.025, *P* = 0.89).

Binary logistic regression analysis suggested that a higher AAT serum level in early pregnancy could increase the odds of SPTB (Table 3). After adjustment, the likelihood of SPTB was associated with higher serum AAT levels when including all samples and samples from the first trimester (Table 3). The multivariable analysis suggested that AAT levels had no association with the probability of SPTB in the second trimester (Table 3). Generally, serum CRP levels did not associate with the odds of SPTB (Table 3). Serum CRP seemed to associate with the odds of SPTB in the univariable analysis but after adjusting for serum AAT, no association was observed (Table 3).

**Table 3:** Binary logistic regression of the effect of serum AAT and serum CRP levels on spontaneous preterm birth interpreted as crude and adjusted odds ratio with 95% confidence intervals.

Study population 1	Univariable analysis			Multivariable analysis		
Predicting variable	cOR	95% CI of cOR	*P* value	aOR	95% CI of aOR	*P* value^a^
The first and second trimester (all samples)						
Serum AAT level (g/l)	2.49	1.53–4.05	< 0.001	2.39	1.41–4.04	0.001
Serum CRP level (mg/l)	1.02	0.99–1.06	0.137	1.00	0.97–1.04	0.885
The first trimester						
Serum AAT level (g/l)	2.02	1.16–3.53	0.014	2.03	1.12–3.66	0.019
Serum CRP level (mg/l)	1.00	0.97–1.05	0.825	0.99	0.95–1.03	0.628
The second trimester						
Serum AAT level (g/l)	4.05	1.30–12.58	0.016	2.87	0.74–11.22	0.129
Serum CRP level (mg/l)	1.08	1.00–1.16	0.047	1.04	0.95–1.12	0.410

*AAT* alpha-1 antitrypsin, *CRP* C-reactive protein, *cOR* crude odds ratio, *CI* confidence interval, *aOR* adjusted odds ratio.

^a^Adjusted for serum AAT and serum CRP.

## Discussion

Previously, we observed that expression of placental AAT is decreased in SPTB^6^. This notion led us to examine AAT levels in maternal serum in relation a pregnancy outcome: SPTB or term birth. We observed higher maternal serum AAT levels in those who did eventually gave birth prematurely. However, a substantial overlap was observed in AAT concentrations between the SPTB and control groups.

A few previous studies have assessed proteomes of maternal sera in early pregnancy and determined differentially expressed proteins between SPTBs and term births. Proteomics of sera from the first trimester of pregnancy have suggested several differentially expressed proteins in SPTB compared to term controls^18^. In their primary analysis, protein expression of AAT was higher in SPTB, but this was not verified using western blot in a larger cohort^19^. In our current study, we observed that serum AAT levels were higher in early pregnancy serum samples from participants with subsequent SPTB. As overlap in the AAT concentrations between the two groups was evident, maternal serum AAT per se cannot constitute a clinically relevant test to predict SPTB.

Another proteomic study utilizing serum from the first trimester of pregnancy revealed several differentially expressed proteins in SPTB^14^. Among these proteins was CRP which was upregulated in SPTB. Different results regarding elevated maternal serum CRP levels and SPTB have been reported, however. Associations between increased serum CRP levels in early pregnancy and pregnancy complications, such as preterm delivery^15,17^, preeclampsia, and intrauterine growth restriction^20^, have been reported. Albeit CRP has not proven to be accurate test to predict risk of SPTB^4^. Larsson et al.^12^ estimated the lower and upper limits of several acute phase proteins, including serum AAT and CRP, during normal pregnancy. They showed that AAT concentration did increase especially between weeks 34–38, whereas CRP had highest values during weeks 28–31. We examined correlations between serum AAT and CRP in both our study populations. A weak to modest positive correlation was suggested in early pregnancy, particularly in the second-trimester samples from the SPTB group. In the control group, correlations in different pregnancy trimesters remained rather weak. As infection and inflammation are associated with SPTB^3^, an acute phase response may be activated already in early pregnancy, leading to increased concentrations of maternal serum AAT and CRP. Additionally, labor comprises several inflammatory factors^3^, and as an acute phase protein, it would be expected that AAT levels were higher during active labor than in elective cesarean section. However, we did not observe a statistically significant difference in serum AAT levels between SPTBs and elective preterm births.

Contrasting case reports have been published about the potential association between low serum AAT levels and the risk of preterm birth^21,22^. One case report suggested that the recurring preterm births may be linked to AAT deficiency^21^, while another reported that a patient with AAT deficiency carried the pregnancy to term^22^. Baron et al.^23^ studied AAT levels in serum collected at admission from women with preterm and term labors, as well as in cases with prelabor rupture of the fetal membranes (PROM) and preterm PROM (PPROM). They did not find a statistically significant difference in maternal AAT concentrations or in AAT antiprotease activity between preterm and term labors and between the PPROM and PROM cases. However, they reported decreased serum AAT levels in two out of 15 PPROM cases and speculated that AAT deficiency could contribute to PPROM. We did not observe a difference in maternal AAT serum levels between preterm and term pregnancies at labor, which is in concordance with the previous result^23^. In our data, PPROM and PROM cases were not distinguished.

Previously, we observed that AAT is present in the extracellular matrix in the decidua and in villous trophoblasts in preterm and term placentas^6^. Human amnions have been demonstrated to have expression of AAT at both mRNA and protein level^24^. However, it is unclear whether AAT in the placenta originates from the placental cells, the mother, and/or the fetus. In the current study, we observed that maternal serum AAT levels in early pregnancy and during labor do not decrease in pregnancies complicated by SPTB. This suggests that AAT production could be locally suppressed in the placenta. It remains to be studied whether increase in maternal serum AAT could act as a compensation mechanism for the downregulation of placental AAT^6^. In the future, a longitudinal cohort, studying serum AAT levels throughout pregnancy could further clarify the relationship between serum AAT and preterm pregnancies. Furthermore, the interactions between maternal and fetal AAT pools remain to be studied.

This ancillary study had certain limitations. One of them is the lack of clinical data, as the present investigation relies on the available information in birth diaries. For example, in study population 1, there was no information about possible maternal infection at the time of outpatient visit for pregnancy-based population screening. However, we studied whether other available, possible confounding factors associate with serum AAT levels. In study population 1, we observed that smoking during pregnancy and in vitro fertilization were more common among women who gave birth prematurely. Smoking did not associate with serum level of AAT in our population of pregnant women, which aligns with previous result from a population of nonpregnant participants^25^. Likewise, we observed that AAT levels did not differ between those who received in vitro fertilization treatments and those who did not. In addition, cervical length measurements were not available in birth diaries. Short cervical length, besides history of preterm birth, is one of the predictors of SPTB. However, routine screening for short cervix was not recommended in present population^26,27^. The sample size during the second half of pregnancy was limited and remains to be studied in the future. The SPTB group likely overrepresented a history of previous preterm births, as the previously reported study focused on mothers having more than one SPTB^28^.

A recent population-based cohort study found that women with diagnosed AAT deficiency had increased risk of preterm birth^29^. In general, genetic AAT deficiency is considered rare^22^. However, underdiagnosis of AAT deficiency is plausible as women may be asymptomatic during their childbearing age while the symptoms appear later in life^29^. It has been estimated that even 90% of patients with severe AAT deficiency may remain undiagnosed^30^. Therefore, it would have been valuable to determine *SERPINA1* (the gene that encodes AAT) genotypes in our current study. Unfortunately, we do not have available DNA samples of the participants in our study populations. Therefore, we cannot determine how much genetic AAT deficiency affects our results. Additionally, the serum concentration of AAT may falsely ascribe to normal levels though the antiprotease capacity of the protein may be decreased^9^. In the future, it is of interest to investigate further how different *SERPINA1* genotypes affect pregnancy outcomes.

The present investigation has several strengths. The participants in study population 1 passed several exclusion criteria, and mothers with major risk factors, such as multiple gestation, maternal diseases that could influence the timing of birth, and alcohol/narcotic use, were excluded^28,31^. In addition, the sample size in study population 1 is adequate.

To summarize, our results showed an association between higher maternal AAT levels in early pregnancy and the risk of SPTB. However, the accuracy of maternal serum AAT in predicting SPTB is weak and it does not constitute as a predictive test of the risk of subsequent SPTB at least by itself. More studies are required to understand the causes and consequences of the maternal–placental–fetal interaction of AAT and its relationship to premature birth.

## Methods

### Ethics statement

Written informed consent was obtained from all study participants. Collection and use of biological samples and processing of patient information were approved by the regional medical research ethics committee of the Wellbeing services county of North Ostrobothnia (79/2003, 14/2010, and 73/2013; amendments). The use of the serum samples from the Finnish Maternity Cohort was approved by the Biobank Borealis scientific committee (BB22-0093). The present study was carried out in accordance with the Declaration of Helsinki.

### Serum samples from the first and second trimesters of pregnancy (study population 1)

In this study, we had two sets of study populations. The first study population (study population 1) comprised women who eventually gave birth preterm (SPTB group) or term (control group). The design of the present study is an ancillary study of a previous prospective study. The participants (*n* = 681) were enrolled for studies from 1998 to 2014 at Oulu University Hospital, Oulu, Finland, or at Tampere University Hospital, Tampere, Finland^28,31^. The exclusion criteria have been described in detail previously^28,31^. The threshold of gestational age (GA) for preterm birth was ≤ 36 + 0 gestational weeks and for term birth from 38 + 0 to 42 + 0 gestational weeks. In preterm births, only labors with spontaneous onset were included. In term births, also labors without spontaneous onset were included.

Corresponding serum samples of the study participants (*n* = 681) were then obtained from the Finnish Maternity Cohort (FMC) serum collection (Biobank Borealis of Northern Finland, Oulu University Hospital, Finland). FMC is a population-based national collection of serum samples that were taken during pregnancy usually between 10 and 14 weeks of gestation. It includes approximately 2 million pregnancies between 1983 and 2016^32^. The FMC includes over 90% of all pregnant Finnish women during this period. The samples were collected at maternity care units for routine screening of congenital infections and the remaining samples (1–3 ml) were stored at − 25 °C for research purposes. Of the 681 samples obtained, following samples were omitted: samples collected before 2001 (*n* = 131), birth date or GA at delivery missing (*n* = 11), miscarriage (*n* = 1), sampling at ≥ 27 weeks of pregnancy (*n* = 2), and medically indicated preterm labor (i.e., elective and emergency cesarean sections without signs of active labor, *n* = 7). After exclusion, a total of 529 serum samples were used in this study, and the samples were collected from 2001 to 2014.

Thresholds for pregnancy trimesters were as follows: GA < 13 + 0 gestational weeks for the first trimester and GA from 13 + 0 to 26 + 6 gestational weeks for the second trimester. Additional clinical information about the participants, such as previous preterm birth, length of gestation at birth, and birth date, was collected retrospectively from birth diaries.

### Serum samples from pregnant women at labor and from nonpregnant controls (study population 2)

The second study population (study population 2) included pregnant and nonpregnant participants. The study participants were prospectively enrolled from 2019 to 2021 at Oulu University Hospital, Oulu, Finland, for a small cohort of SPTBs, elective preterm births, and term births. Serum was collected from pregnant women at admission in the delivery room or the operating room. Then, the sample was assigned into a group based on the delivery type and GA at labor. The groups were SPTB (*n* = 8), elective preterm birth (*n* = 4) and term birth (*n* = 20). The threshold for GA was < 37 + 0 gestational weeks for preterm births. There were no signs of spontaneous onset of labor in the elective preterm births, and the baby was delivered by emergency or elective cesarean section. GA ranged from 37 + 4 to 41 + 0 gestational weeks in term birth cases. Only singleton pregnancies were included. Relevant clinical data about the pregnancies were collected retrospectively from the birth diaries, such as length of pregnancy. For nonpregnant controls, serum was collected from healthy women (age from 21 to 41 years, *n* = 15) who were not pregnant at the time of sample collection. The absence of pregnancy was not confirmed with a pregnancy test.

### Sample preparation and analysis of serum protein levels

The samples from study population 1 were analyzed for AAT and CRP at HUSLAB Laboratory, Helsinki, Finland, and 150 μl of serum was diluted with 350 μl of 0.9% NaCl. The samples of study population 2 were analyzed as follows: AAT at HUSLAB Laboratory, Helsinki, Finland, and CRP at Nordlab Laboratory, Oulu, Finland. These samples were diluted 1:1 with 0.9% NaCl. The methods for AAT and CRP measurements are photometric, immunochemical, and accredited at the HUSLAB Laboratory. CRP measurement at the Nordlab Laboratory is immunonephelometric, and accredited. The detection limit of serum AAT is 0.05 g/l and for serum CRP 0.16 mg/l.

### Statistical analyses

Baseline characteristics are expressed as medians and numbers with suitable descriptive statistics. Statistical differences between the SPTB and control groups were calculated with appropriate statistical tests. Missing values were excluded pairwise. AAT levels in study population 1 are represented as the mean and standard deviation (SD) as the data were normally distributed. Student’s *t*-test was used to determine the statistical significance between two groups. In study population 2, AAT levels are interpreted as the median and interquartile range (IQR) as the data were skewed, and the Mann–Whitney *U* test was used for statistical comparisons (nominal association). Correlation coefficients (*r*_*s*_) between serum AAT and CRP were calculated with Spearman’s rank-order correlation in both study populations. In study population 1, binary logistic regression was performed to investigate the relationship between SPTB, and maternal serum AAT levels taken at the first or second trimester, and in both trimesters combined. Pregnancy outcome was treated as dichotomous variable (SPTB vs. term birth). Serum AAT and serum CRP levels were included as explanatory variables. The effect on the odds ratio (OR) of SPTB is reported as crude OR (univariable analysis) and adjusted OR (multivariable analysis), both with 95% confidence intervals and *P* values. The analyses were performed with SPSS Statistics 28.0 (IBM Corporation, Armonk, NY). In all statistical analyses, a *P* value of < 0.05 was considered as statistically significant.

### Supplementary Information

Supplementary Information.

## Data Availability

The datasets generated during and/or analysed during the current study are available from the corresponding author on reasonable request.
