# Supplementary material for: Maternal serum alpha-1 antitrypsin levels in spontaneous preterm and term pregnancies
Source: Sci Rep. 2024 May 11;14:10819. doi: 10.1038/s41598-024-61206-z (PMC11088650; doi:10.1038/s41598-024-61206-z)
Supplement: Supplementary file 1 — Supplementary Information. [file 41598_2024_61206_MOESM1_ESM.pdf]

SUPPLEMENTARY FILE

Supplementary Table S1. Effect of different pre-pregnancy variables on maternal serum AAT levels in study population 1.

**Variable**		**Serum AAT level, mean (SD), g/l**		***P*-value**[a]
In vitro fertilization or treated with hormones		Yes	No	
	Whole population	1.59 (0.40)	1.56 (0.44)	0.774
	Only SPTBs	1.77 (0.39)	1.68 (0.47)	0.583
	Only controls	1.33 (0.28)	1.52 (0.41)	0.259
Smoking during pregnancy		Yes	No	
	Whole population	1.58 (0.42)	1.53 (0.42)	0.459
	Only SPTBs	1.60 (0.60)	1.67 (0.42)	0.602
	Only controls	1.57 (0.27)	1.49 (0.43)	0.415
Age of the mother at sampling		< 40 years	≥ 40 years	
	Whole population	1.56 (0.44)	1.63 (0.37)	0.392
	Only SPTBs	1.67 (0.46)	2.01 (0.39)	0.102
	Only controls	1.52 (0.42)	1.54 (0.30)	0.812

[a]*P*-value calculated with Student's *t* test.

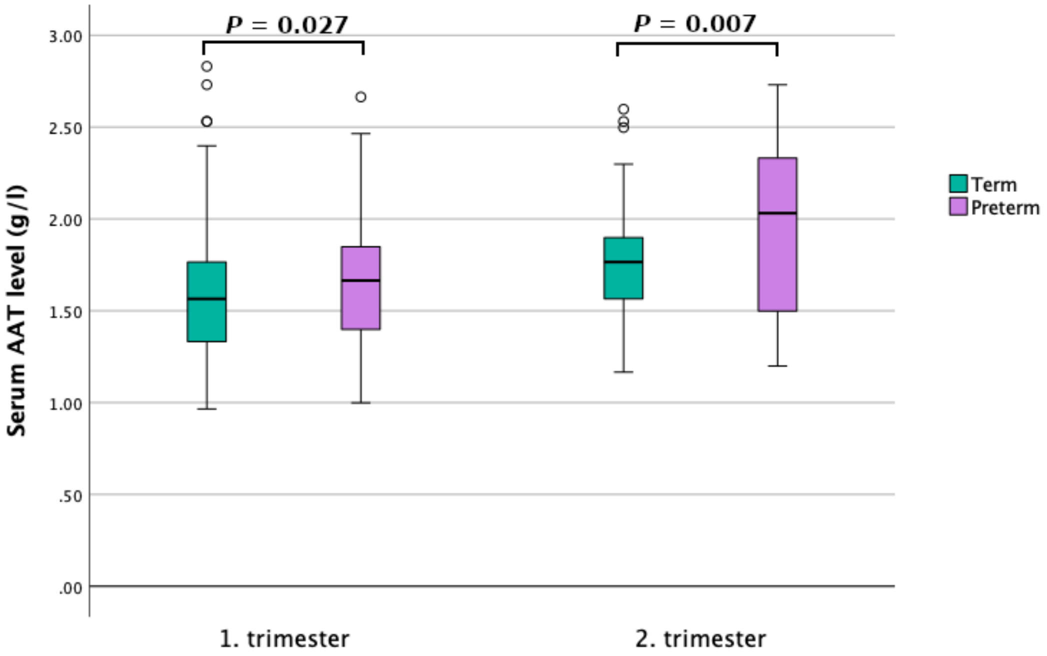

Supplementary Fig. S1. Maternal serum AAT levels in study population 1 after exclusion of those with serum AAT levels of < 0.96 g/l. Band indicates the median, interquartile range (IQR) represented by box and 1.5*IQR by whiskers.

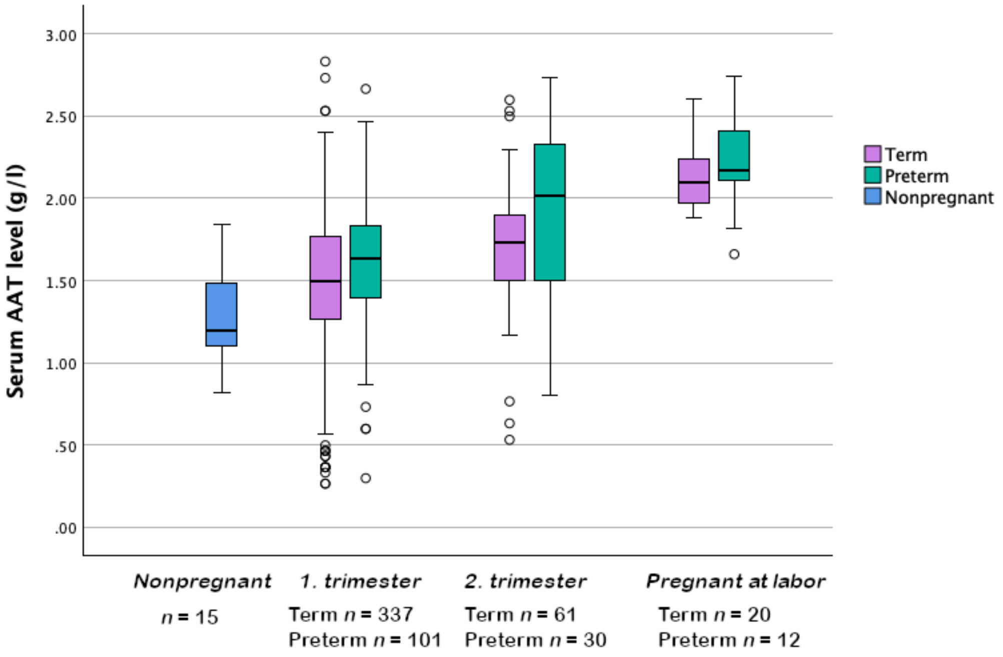

Supplementary Fig. S2. Maternal serum AAT levels, including both study populations: samples from nonpregnant women (study population 2), samples from the first trimester (study population 1, SPTBs and term births), samples from the second trimester (study population 2, SPTBs and term births), and pregnant women at labor (study population 2, preterm births [SPTBs, elective preterm births], and term births). Band indicates the median, interquartile range (IQR) represented by box and 1.5*IQR by whiskers.

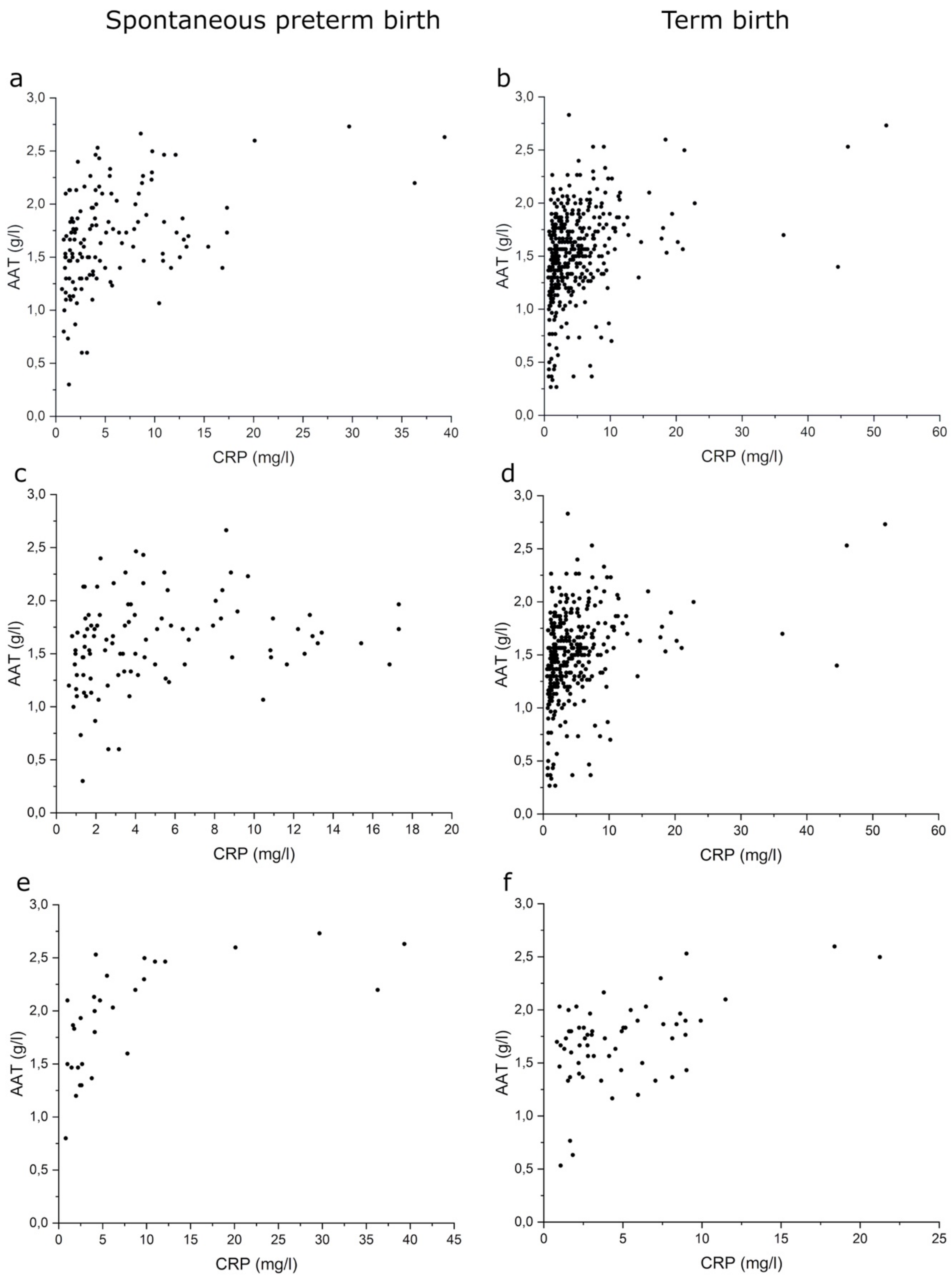

Supplementary Fig. S3. Relationship between maternal serum alpha-1 antitrypsin (AAT) and C-reactive protein (CRP) levels in spontaneous preterm birth and term birth in early pregnancy (study population 1). Spontaneous preterm and term birth cases are indicated by a circle. (a) and (b) Correlation in samples from the first and second trimesters. (c) and (d) Correlation in samples from the first trimester, and (e) and (f) correlation in samples from the second trimester.
